# Delay in diagnosing Kawasaki disease. Identifying the root cause at the referral base of a regional children’s hospital

**DOI:** 10.1186/1546-0096-10-S1-A91

**Published:** 2012-07-13

**Authors:** Daniela Vidal, Lorena Mariana Franco, Maria Elena Rama, Maria Teresa Apaz, Carlos D Rose

**Affiliations:** 1 DuPont Hospital for Children, Wilmington, DE, USA; 2 Hospital de Ninos, Cordoba, CO, Argentina

## Purpose

To establish the frequency of coronary artery disease in our KD population and to investigate its correlation with the time of institution of IV-Ig therapy. In addition we hope to establish if the time to institution of therapy is related to delay in diagnosis/referral or timely availability of IV-Ig supply at our institution.

## Methods

Retrospective medical record review of a cohort of patients with KD over a 10 year period. All patients who met diagnostic criteria of KD seen in our Institution between January of 1999 and January of 2009 were included. The patient population was segregated into two groups according to the presence or absence of coronary aneurisms (CA + and CA -). Days from symptom onset to diagnosis and days from symptom onset to infusion were recorded. Demographic, clinical, echocardiographic and laboratory variables were recorded and described as well. Descriptive statistics and Mann-Whitney Rank Sum Test for comparison of groups were applied. A p ≤ 0.05 was considered significant.

## Results

A hundred eleven patients were enrolled, 92 had complete records. Mean age at onset was 41.3 months. M/F ratio 1.4. Twenty three patients showed some degree of cardiac involvement of which 11 (12%) consisted of CA. The CA + group received first dose of IV-Ig 14,7 +/- 7,2 days from onset of symptoms, while the CA – group received IV-Ig 10,35 +/- 8,2 days (p= 0,026). For the CA+ group time to diagnosis was 13,7 +/- 7,55 days while it was 9,6 +/- 7,51 days in the CA - group (p= 0,04). The time from diagnosis to IV-Ig therapy was similar in the two groups (p= 0.465) suggesting no supply difficulties at our institution.

## Conclusion

Despite the efficacy of IV-Ig in preventing CA in KD our frequency of CA remains high at 12%. Delay in institution of therapy (all patients eventually received IV-Ig therapy) is the suspected underlying cause. This delay seems to be related to either access barriers or timely diagnosis. A referral bias towards more severe cases cannot be ruled out by the study. Educational efforts to improve index of suspicion as well as improvement of access are paramount problems to be addressed by decision makers if the frequency of potentially permanent coronary artery damage is to be reduced among children with KD in our region.

## Disclosure

Daniela Vidal: None; Lorena Mariana Franco: None; Maria Elena Rama: None; Maria Teresa Apaz: None; Carlos D. Rose: None.

